# Compact FPGA hardware architecture for public key encryption in embedded devices

**DOI:** 10.1371/journal.pone.0190939

**Published:** 2018-01-23

**Authors:** Luis Rodríguez-Flores, Miguel Morales-Sandoval, René Cumplido, Claudia Feregrino-Uribe, Ignacio Algredo-Badillo

**Affiliations:** 1 Department of Computer Science, Instituto Nacional de Astrofísica, Óptica y Electrónica, Puebla, 72840 Mexico; 2 Cinvestav-Tamaulipas, Victoria City, 87130 Mexico; 3 Department of Information Technology in the Polytechnic University of Tlaxcala, Tlaxcala, Mexico; University of Texas at San Antonio, UNITED STATES

## Abstract

Security is a crucial requirement in the envisioned applications of the Internet of Things (IoT), where most of the underlying computing platforms are embedded systems with reduced computing capabilities and energy constraints. In this paper we present the design and evaluation of a scalable low-area FPGA hardware architecture that serves as a building block to accelerate the costly operations of exponentiation and multiplication in GF(p), commonly required in security protocols relying on public key encryption, such as in key agreement, authentication and digital signature. The proposed design can process operands of different size using the same datapath, which exhibits a significant reduction in area without loss of efficiency if compared to representative state of the art designs. For example, our design uses 96% less standard logic than a similar design optimized for performance, and 46% less resources than other design optimized for area. Even using fewer area resources, our design still performs better than its embedded software counterparts (190x and 697x).

## Introduction

With the coming of Ubiquitous Computing [1], the Internet of Things (IoT) [2], and Wearable Computing [3], it is expected that electronic devices in the form of embedded systems acquire, store, process and communicate sensitive data in industrial sectors such as the medical, surveillance, nuclear, and defense, to mention some examples. Security in these embedded and networked devices has become critical, and currently it is one of the main aspects delaying the deployment of pervasive computing environments [4]. Some suppliers are addressing system security at the component level, with encryption being one of the most effective ways to provide the security services of authentication, integrity and confidentiality [5, 6].

It is hard for some security schemes such as those based on public key cryptography to achieve high throughput without the help of hardware modules [7]. That is why the development of hardware cryptographic modules for security is an active area of research. Some microcontrollers found in current embedded devices, for example Atmel and Microchip, now include hardware encryption/decryption engines, which demonstrates the support for hardware encryption/decryption not only for 32-bit processors, but also for 8-bit and 16-bit. Atmel has chosen to integrate the public key cryptographic security protocols ECDH (key agreement protocol) and ECDSA (sign-verify digital signatures for authentication) on its ATECC508A encryption chip [8]. In another example, Infineon has added secure authentication protocols to a power module designed for IoT applications, such as internet-connected industrial drives. For example, its MIPAQ Pro power module incorporates a security microcontroller to provide authentication of original parts [9].

The security services provided by means of public key cryptographic algorithms [10–12] demand a large number of arithmetic operations on abstract algebraic structures (finite fields and groups), usually executed over large numbers (160–3072 bits), which makes them considerably time-consuming operations. This situation has motivated the creation of specialized hardware with faster computation as the main design goal, which comes at the cost of a high consumption of hardware resources [13, 14].

However, the computing constraints of embedded systems demand implementations of cryptographic modules using fewer area resources [15]. This implementation approach is considered in the research field of lightweight cryptography [15]. Although that research has mainly focused on private key algorithms and cryptographic primitives [16], the one for public key cryptography has been recently increasing [17].

For hardware realization of algorithms Field Programmable Gate Arrays (FPGAs) could be preferred because of flexibility, low cost, fast time to market, and long-term maintenance [18]. Particularly for cryptographic applications, FPGAS have the advantage that the hardware design can be re-configured or reprogrammed whenever a new security requirement is necessary or when the algorithm must be adapted to support higher security levels [19]. Today FPGAs are not only used as rapid prototyping devices but as final products [20]. Moreover, by providing on-chip integration of processors and co-processors, FPGAs are now becoming a preferred platform for System-on-Chip (SoC). Low-cost and low-power FPGAs are available in the market, and it is expected they become popular for applications such as wireless sensor network (WSN) or the Internet of Things (IoT) [21]. For example, Xilinx and Digilent promoted the MicroZed Industrial IoT Starter Kit which is based on Zynq-7000 Programmable SoC. Since FPGAs are considered as final implementation devices it is desirable to integrate many functionalities in the same FPGA, in which cryptographic modules are only a part of an entire system [22].

Nowadays, modern FPGAs have a large amount of programmable logic components and some resources on chip, such as Digital Signal Processing (DSPs) and Block Rams (BRams) [23]. If FPGAs are the implementation technology, all resources on chip (DSPs, BRams, etc.) are available even if they are not used. However, a substantial amount of power can be saved using embedded blocks instead of programmable logic [24]. Furthermore, embedded blocks are smaller and have between 5x and 12x lower power than equivalent programmable logic implementations [24]. The use of DSPs and BRams in FPGA-based cryptography hardware architectures could contribute to save standard configurable logic for implementing other system components and also to save power consumption [25].

This work focuses on a low area FPGA-based hardware construction for the main and most time-consuming operation in the standardized public key cryptosystems RSA [10], DSA [12] and DH [26]: the exponentiation operation in GF(p). The use of DSPs and BRams in the FPGA is exploited to reduce the reconfigurable logic. Other related works have also considered this design strategy [25, 27, 28]. Our approach is to first create novel digit-digit based arithmetic algorithms in GF(p) that favor the design of the corresponding hardware architectures. Under this approach, the multiplier, multiplicand and modulus are partitioned and processed in digits of *k* bits, similar to a software approach except that parallelism is exploited.

We show in this work that the digit-digit approach allows low-area hardware designs for exponentiation in GF(p) without loss of efficiency, while keeping the advantages of a customized but flexible hardware module suitable for encryption in embedded devices. These advantages come with the property of scalability, thus supporting exponentiation over any field GF(p) with the same datapath, which only depends on the size *k* of the digit and not on the size of *p*.

The main contributions in the present paper are the design of a novel Montgomery multiplication algorithm, its corresponding low-area hardware architecture, and a low-area hardware architecture for the Montgomery Powering Ladder (MPL) for GF(p) exponentiation that uses two hardware modules of the Montgomery multiplier as main building blocks. The main advantage of the design of the MPL hardware architecture is that all the data of the operands and temporary values are mapped to external memory blocks, so the datapath complexity is reduced. These memory blocks are used as both input and output sources thus maximizing their utilization. For example, the same memory block is used for storing input operand and the partial results during the execution of the algorithm. That is, at the beginning of a clock cycle one digit *d*_*i*_ is read and used as input parameter in the multiplier to obtain the digit *r*_*i*_ of the partial result. At the end of the same clock cycle, *r*_*i*_ is stored in the same memory block replacing *d*_*i*_. As main distinctives, the hardware architecture for exponentiation in GF(p) proposed in this paper:

- Has as main goal low area instead of high performance,
- Implements a datapath based on the digit size instead of the operands size,
- Stores all operands and partial results in memory blocks,
- Is scalable, the same datapath could be used to compute modular exponentiation for different operand sizes since datapath is based on the digit size, not in the operand size,
- The efficiency is not lost even using fewer area resources.

The results obtained from a wide experimental evaluation reveal specific configurations {operand-size, digit-size} that lead to lower-area designs as well as more efficient designs, or with better performance if compared to related works.

The rest of this paper is organized as follows: Section Exponentiation in GF(p) reviews the operations of multiplication and exponentiation in GF(p). Section Proposed Method presents the digit-digit computation approach for multiplication and exponentiation in GF(p), and the design details of the proposed hardware architectures. Section Implementation Results provides details about the experimentation, describes the implementation results, and provides comparisons. Finally, section Conclusion summarizes the contributions of this work and gives directions for future work.

## Exponentiation in GF(p)

The finite field GF(p) with *p* a prime number is defined as the set of integers {0, 1, …, *p* − 1} together with the operations of addition and multiplication modulo *p* [29]. Exponentiation in GF(p) is defined as *g*^*e*^ mod *p* with g∈GF(p) and e∈N. The basic method for exponentiation by multiplying *g* by itself *e* − 1 times is totally inefficient. Faster algorithms have been proposed to compute *g*^*e*^, one of the most used nowadays is the Montgomery Powering Ladder method [30].

### The Montgomery Powering Ladder algorithm (MPL)

The MPL algorithm was originally proposed as a way to speed up the scalar multiplication in the elliptic curve domain [30]. Later, Joe and Yen [31] extended its scope to execute exponentiation in an abelian group. The main advantages of MPL are that it does not have conditional jumps nor extra operations, as in other approaches, which makes it resistant to certain kind of side channel attacks, such as the Simple Power Analysis (SPA) attack [32]. The MPL method for GF(p) exponentiation is listed in Algorithm 1. It is assumed that the exponent *e* is *L* bits in size, and *e*_*i*_ is the *i*th bit of *e*.

**Algorithm 1** MPL method for exponentiation in GF(p)

**Require**: g∈GF(p), $e={\left(e_{L-1},\cdots ,e_{0}\right)}_{2}\in N$ and *p* a prime number defining GF(p)

**Ensure**: *g*^*e*^ mod *p*

1: *R*0 ← 1; *R*1 ← *g*;

2: **for** *i* = *L* − 1 downto 0 **do**

3:  **if** *e*_*i*_ = 1 **then**

4:   *R*0 ← *R*0 × *R*1 mod *p*;

5:   *R*1 ← *R*1 × *R*1 mod *p*;

6:  **else**

7:   *R*0 ← *R*0 × *R*0 mod *p*;

8:   *R*1 ← *R*1 × *R*0 mod *p*;

9:  **end if**

10: **end for**

11: **return** *R*0;

The crucial operation in the MPL algorithm is GF(p) multiplication. One of the most used algorithms for efficient multiplication in GF(p) is the Montgomery method [33]. This algorithm employs only simple addition, subtraction and shift operations to avoid trial division by the modulus *p*, which is very expensive in hardware implementations.

### Montgomery multiplication

The Montgomery multiplication algorithm [33] (MMA) listed in Algorithm 2 has been used as a foundation for diverse implementations of modular multiplication. Given two numbers A,B∈GF(p), they are first transformed to the Montgomery domain by doing *A*′ = *A* × *R* mod *p* and *B*′ = *B* × *R* mod *p*. *A*′ and *B*′ are called Montgomery numbers. MMA uses *A*′, *B*′ together with a number *R* such that gcd(*p*, *R*) = 1. Here, *p* is an *N*-bit integer number with 2^*N* − 1^ ≤ *p* < 2^*N*^. It is common to use *R* = 2^*N*^. Based on this fact, it is possible to compute the numbers *R*^−1^ and *p*′ using the identity *R* × *R*^−1^ + *p* × *p*′ = 1, with 0 < *R*^−1^ < *p* and 0 < *p*′ < *R*, using methods such as the extended Euclidean Algorithm. The Montgomery product is defined as *A*′ × *B*′ × *R*^−1^ mod *p*.

**Algorithm 2** Montgomery multiplication algorithm (**MMA**)

**Require**: Integers *A*′, *B*′, *R* = 2^*N*^, and *p* a *N*-bit prime number.

**Ensure**: *A*′ × *B*′ × *R*^−1^ mod *p*

1: *t* ← *A*′ × *B*′

2: *q* ← (*t* mod *R*) × *p*′ mod *R*

3: *u* ← (*t* + *qp*)/*R*

4: **if** *u* ≥ *p* **then**

5:  *u* ← *u* − *p*

6: **end if**

7: **return** *u*;

The transformation of *A* to *A*′ and viceversa can be done using the MMA algorithm, since *A*′ = MMA(*A*, *R*^2^), and *A* = MMA(*A*′, 1).

Thus, one modular multiplication *A* × *B* mod *p* in GF(p) requires to compute the next four MMA multiplications:

$$\begin{array}{ccc} A^{\prime } & = & \text{MMA}(A,R^{2})\\ B^{\prime } & = & \text{MMA}(B,R^{2})\\ Z^{\prime } & = & \text{MMA}(A^{\prime },B^{\prime })\\ C & = & \text{MMA}(Z^{\prime },1). \end{array}$$

The additional operations for number conversion, together with the additional computation of *p*′, makes the Montgomery method inefficient for computing a single multiplication in GF(p) if compared with traditional multiplication algorithms.

However, the Montgomery algorithm is significantly faster when many consecutive multiplications are required, such as in a GF(p) exponentiation (see Algorithm 1). In this case, domain conversion is needed only at the beginning and at the end of the cumulative multiplications.

## Proposed method

### Digit-digit GF(p) exponentiation algorithm

The notation used from here on is shown in Table 1. Let *X*, *Y* be numbers in GF(p). Using the radix *β* = 2^*k*^, the digit-based representation of *X*, *Y* is defined as in Eq 1.

**Table 1:** Notation.

Symbol	Description
*N*	Operand size in bits
*n*	Total *k*-bit digits of operands
*p*	The modulus defining GF(p)
*X*, *Y*, *A*	Elements in GF(p)
*β*	Radix *β* = 2^*k*^
*p*′	Precomputed value, *p*′ = −*p*^−1^ mod *β*
*Z*_*i*_	The *i*th digit of element Z∈GF(p)
*e*	Exponent
*e*_*i*_	The *i*th bit of exponent *e*
*L*	Exponent size in bits
*X*^<*i*>^	Value of *X* at iteration *i*

$$\begin{array}{c} X=\sum_{i=0}^{n-1}X_{i}\beta^{i},\;Y=\sum_{i=0}^{n-1}Y_{i}\beta^{i}\\ X_{i},Y_{i}\in \left\{0,1,\ldots ,\beta -2,\beta -1\right\} \end{array}$$ (1)

Lets define *MMD*(*X*, *Y*, *p*) as the function that computes the Montgomery product of *X*, *Y*, processing them internally in a digit-by-digit fashion. With the previous notation, Algorithm 1 can be transformed into Algorithm 3, where the exponentiation operation *g*^*e*^ mod *p* is computed using a digit-by-digit processing. In that algorithm, it is assumed that both *g* and *g*^*e*^ are in the Montgomery domain. The exponent *e* is expressed in the same way than in Algorithm 1, but ‘1’ must be treated as a Montgomery number, that is, it must be transformed to 1 × 2^*N*^ mod *p*.

**Algorithm 3** Digit-digit MPL algorithm

**Require**: *e* = (*e*_*L* − 1_, ⋯, *e*_0_)_2_, $g=\sum_{i=0}^{n-1}g_{i}\beta^{i}$, *p*

**Ensure**: $C=\sum_{i=0}^{n-1}C_{i}\beta^{i}=\left(g^{e}\right)\times R$ mod *p*

1: *X* ← 1 × 2^*N*^ mod *p*;

2: *Y* ← *g*;

3: **for** *i* = *L* − 1 downto 0 **do**

4:  **if** *e*_*i*_ == 1 **then**

5:   *X* ← *MMD*_0_(*X*, *Y*, *p*);

6:   *Y* ← *MMD*_1_(*Y*, *Y*, *p*);

7:  **else**

8:   *X* ← *MMD*_0_(*X*, *X*, *p*);

9:   *Y* ← *MMD*_1_(*Y*, *X*, *p*);

10:  **end if**

11: **end for**

12: **return** *X*;

A direct hardware implementation of Algorithm 3 requires two modules for the *MMD* function, say *MMD*_0_ and *MMD*_1_, which can work in parallel at each iteration. The main advantage of Algorithm 3 is that the *k*-bit digits of operands *X* and *Y* can be stored in *n* × *k* memory blocks, so the hardware realization of the *MMD* function does not require internal logic to store its operands.

However, note that in Algorithm 3 the partial result at iteration *i* become the input data at iteration *i* + 1. That is, the operands’ digits are used and overwritten during the same iteration. So, the main challenge to implement Algorithm 3 without using additional and redundant storage for operands is to design a control logic that correctly parses, accesses and reuses the operands’ digits directly from block memories.

The critical component in Algorithm 3 is the embedded Montgomery multiplier. Some works in the literature have studied and proposed a hardware module for the Montgomery algorithm using a digit-digit approach. The most recent is reported in [34], and could well serve as the *MMD*_0_ and *MMD*_1_ modules required in Algorithm 3. Although the multiplier presented in [34] was developed to be used in cryptography operations such as in RSA cryptosystems, the multiplier as it is could not be useful for constructing a hardware architecture for Algorithm 3. The main reasons are:

- The multiplier in [34] does not take into account that the result of the multiplication is used again as one of the input operands, as it is required in the MPL algorithm. The internal and external dataflow in the multiplier should be redesigned to avoid additional and redundant storage.
- In [34], the partial results at each iteration *i* are stored in a shift register, not in a memory. Thus, additional latency would be required to move the content of the shift register at the end of the main loop in Algorithm 3 to the memory storing the multiplication operands.

The first step in our design methodology was to redesign the Montgomery hardware architecture in [34] in order to have a useful *MMD* module based on the Montgomery multiplier for Algorithm 3. Once having the new digit-digit Montgomery multiplier, the next step in the methodology was to design the novel hardware architecture for GF(p) exponentiation.

### Hardware architecture for digit-digit GF(p) multiplication

Algorithm 4 was presented in [34] for iterative computation of a Montgomery product. In that algorithm, the product is obtained one digit at a time per clock cycle, stored and obtained from a shift register *A* that shifts *k*-bits (one digit) to the right at a time. This shift register represents the variable *A* in Algorithm 4 that stores the partial multiplications at each iteration *i*.

**Algorithm 4** Iterative digit-digit MMA algorithm presented in [34]

**Require**: $X=\sum_{i=0}^{n-1}X_{i}\beta^{i}$, $Y=\sum_{i=0}^{n-1}Y_{i}\beta^{i}$, $p=\sum_{i=0}^{n-1}p_{i}\beta^{i}$, 0 < *X*, *Y* < 2 × *p*, *R* = *β*^*n*^, with *p*′ = −*p*^−1^ mod *β*

**Ensure**: $A=\sum_{i=0}^{n-1}a_{i}\beta^{i}=X\times Y\times R^{-1}$ mod *p*

1: *A* ← 0;

2: **for** *i* ← 0 to *n* − 1 **then**

3:  *c*^<0>^ ← 0

4:  **for** *j* ← 0 to *n* − 1 **do**

5:   *s*^<*j*>^ ← [*A*_0_ + *X*_*j*_ × *Y*_*i*_]

6:   **if** *j* = 0 **then**

7:    *q*^<*i*>^ ← (*s*^<*j*>^ × *p*′) mod *β*

8:   **end if**

9:   *r*^<*j*>^ ← *q*^<*i*>^ × *p*_*j*_

10:   {*c*^<*j*+1>^, *t*^<*j*>^}←*s*^<*j*>^ + *r*^<*j*>^ + *c*^<*j*>^

11:   *A* ← *SHR*(*A*)

12:   *A*_*n*−1_ ← *t*^<*j*>^

13:  **end for**

14:  *A* ← *SHR*(*A*)

15:  *A*_*n*−1_ ← *c*^<*n*>^

16: **end for**

17: **return** *A*;

On the one hand, the Montgomery multiplier in Algorithm 4 delivers the result in a shift register. On the other hand, the input operands for the multiplier reside in memory blocks. This is the main inconvenient when using Algorithm 4 as the *MMD* module for Algorithm 3, because the multiplication result at iteration *i* (stored in a shift register) must be the input data to the multiplier at iteration *i* + 1 (and must reside in a memory block). A shift register—memory block interface would be needed to solve this problem, of course with the associated cost of additional resources and an increased latency.

In the present paper we redesign Algorithm 4 and its corresponding datapath in such a way that the product and partial results in *A* reside in a memory block. The main changes in the dataflow include the control for the read/write operations over *A* in lines 5, 11, 12, 14 and 15 in Algorithm 4. With these changes, the partial Montgomery multiplication at the end of iteration *i*, in Algorithm 3, can be now treated as an input operand at iteration *i* + 1 by multiplexing data ports in the corresponding memory blocks, thus avoiding the introduction of more logic and time overhead.

**Fig 1:**
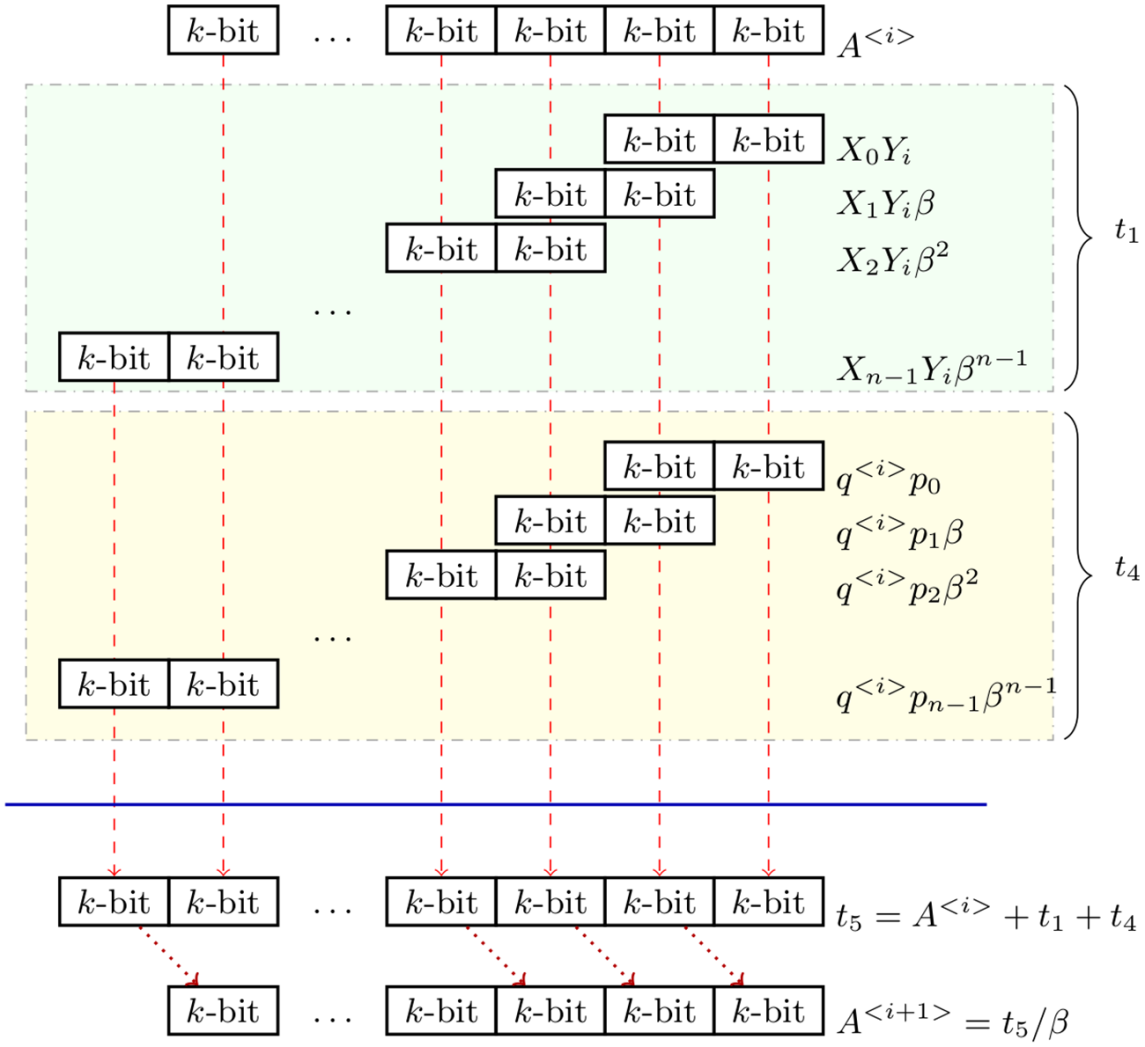
*A*^<*i*+1>^ computation in a digit by digit approach.

Algorithm 4 is based on the Montgomery algorithm proposed by C. Walter [35], Algorithm 5. From a sequential computing approach, the lines 3 and 4 of Algorithm 5 could be performed by the set of operations described in Eq 2. Once *q*^<*i*>^ has been computed, the partial multiplications *t*_1_ = *X* × *Y*_*i*_ and *t*_4_ = *q*^<*i*>^ × *p*, and addition *t*_5_ = *A*^<*i*>^ + *t*_1_ could be performed in a digit by digit fashion. That is, for each iteration *i* in Algorithm 5, *A*^<*i*+1>^ is computed by processing iteratively the digits *X*_*j*_, *A*_*j*_, and *p*_*j*_ from *X*, *A*^<*i*>^, and *p* respectively, thus computing a digit *j* of *A*^<*i*+1>^ at a time (see Fig 1).

**Algorithm 5** Iterative Montgomery Multiplication [36]

**Require**: Integer *X* and *Y*, with 0 ≤ *X*, *Y* < 2 × *p*, *R* = *β*^*n*+1^ with *gcd*(*p*, *β*) = 1, and *p*′ = −*p*^−1^ mod *β*

**Ensure**: *A* = *X* × *Y* × *R*^−1^ mod $p=\sum_{i=0}^{n}A_{i}\beta^{i}$

1: *A* ← 0;

2: **for** *i* ← 0 to *n* **do**

3:  *q*^<*i*>^ ← (*A*_0_ + *X*_0_ × *Y*_*i*_) × *p*′ mod *β*

4:  *A*^<*i*+1>^ ← ([*A*^<*i*>^ + *X* × *Y*_*i*_] + *q*^<*i*>^ × *p*)/*β*

5: **end for**

6: **return** *A*_*n*_;

$$\begin{array}{c} \begin{array}{cc} t_{1} & =X\times Y_{i}\\ t_{2} & =A_{0}+X_{0}\times Y_{i}\\ q^{<i>} & =t_{2}\times p^{\prime }\;\text{mod}\;\beta \end{array}\;\;\begin{array}{cc} t_{4} & =q^{<i>}\times p\\ t_{5} & =A^{<i>}+t_{1}+t_{4}\\ A^{<i+1>} & =t_{5}/\beta \end{array} \end{array}$$ (2)

Fig 1 shows the digit by digit operations for computing *A*^<*i*+1>^ iteratively. At the beginning of iteration *i*, *q*^<*i*>^ is computed. Then, each digit of *A*^<*i*+1>^ is obtained at each next clock cycle *j*. Note that the first digit (always equal to zero) will be discarded at the end of iteration *i* when the operation *t*_5_/*β* executes. So, digits of *A*^<*i*+1>^ must be stored in the corresponding output memory starting from iteration *j* = 2.

Algorithm 6 reflects the modifications to Algorithm 4 needed for computing a digit-digit Montgomery multiplication, well suited to be used in the proposed GF(p) exponentiator.

**Algorithm 6** New iterative Montgomery Multiplication algorithm

**Require**: $X=\sum_{i=0}^{n-1}X_{i}\beta^{i}$, $Y=\sum_{i=0}^{n-1}Y_{i}\beta^{i}$, $p=\sum_{i=0}^{n-1}p_{i}\beta^{i}$, 0 < *X*, *Y* < 2 × *p*, *R* = *β*^*n*^ with *p*′ = −*p*^−1^ mod *β*

**Ensure**: $A=\sum_{i=0}^{n-1}a_{i}\beta^{i}=X\times Y\times R^{-1}$ mod *p*

1: *A* ← 0;

2: **for** *i* ← 0 to *n* − 1 **do**

3:  *c*^<0>^ ← 0

4:  **for** *j* ← 0 to *n* − 1 **do**

5:   *s*^<*j*>^ ← [*A*_*j*_ + *X*_*j*_ × *Y*_*i*_]

6:   **if** *j* = 0 **then**

7:    *q*^<*i*>^ ← (*s*^<*j*>^ × *p*′) mod *β*

8:   **end if**

9:   *r*^<*j*>^ ← *q*^<*i*>^ × *p*_*j*_

10:   {*c*^<*j*+1>^, *t*^<*j*>^}←*s*^<*j*>^ + *r*^<*j*>^ + *c*^<*j*>^

11:   **if** *j* > 0 **then**

12:    *A*_*j*−1_ ← *t*^<*j*>^

13:   **end if**

14:  **end for**

15:  *A*_*n*−1_ ← *c*^<*n*>^

16: **end for**

17: **return** *A*;

The inner loop of Algorithm 6 requires *n* clock cycles. One clock cycle is needed at the beginning to compute *q*^<*i*>^. One clock cycle at the end of the inner loop is necessary to store the last carry, *c*^<*n*>^, in memory *A*, as explained previously. So, the computing of *q*^<*i*>^ (*i* > 0) and the writing of *c*^<*n*>^ can occur during the same clock cycle. Additionally, the output of each memory bank can be pipelined to reduce the critical path at the cost of an extra cycle in the latency. If this is done, the total latency of the hardware module for *MMD* implementing Algorithm 6 requires *n*(*n* + 1) + 4 clock cycles.

**Fig 2:**
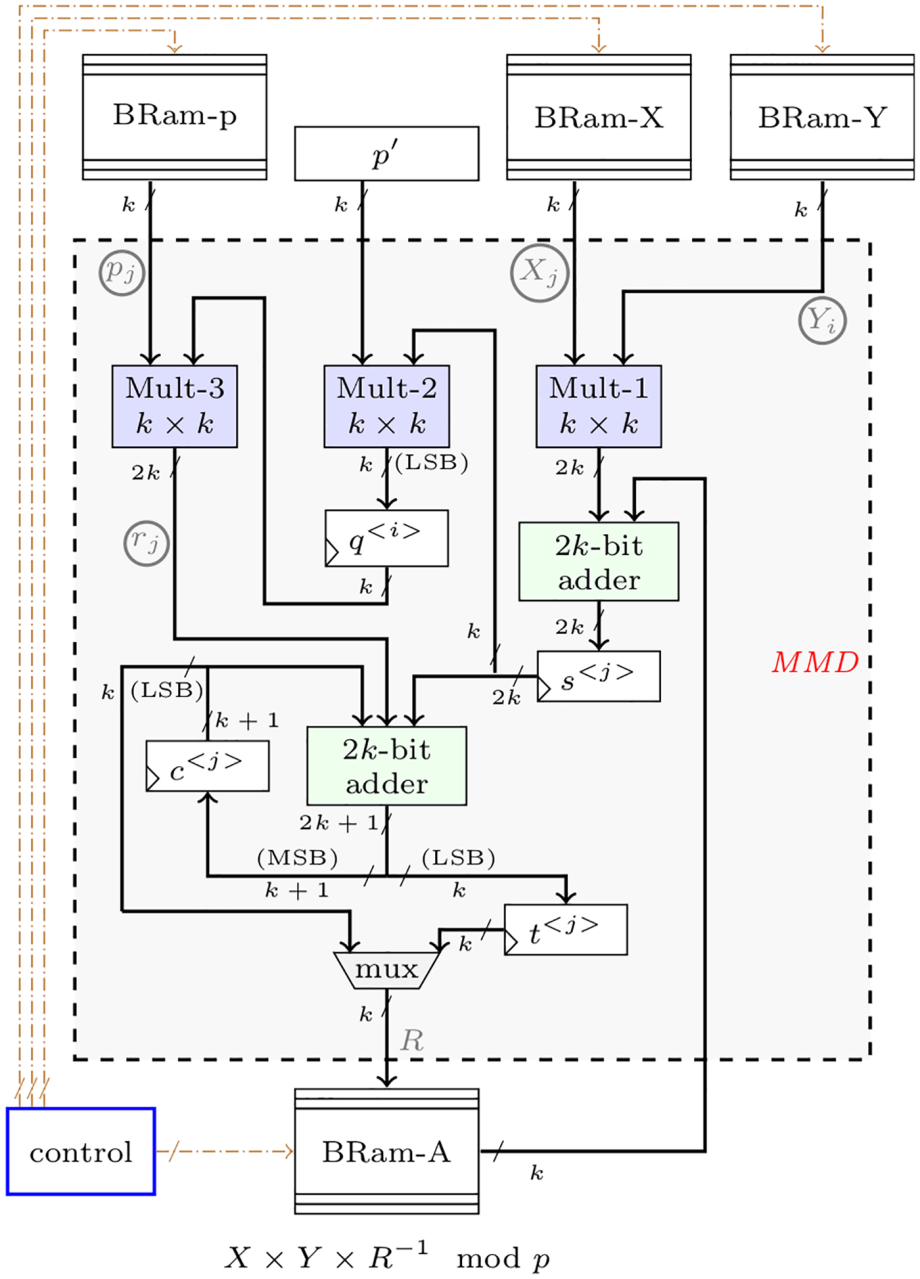
New digit-digit Montgomery multiplier architecture, memory and result reside in memory blocks.

The novel hardware architecture for digit-by-digit Montgomery multiplication is shown in Fig 2. In that figure, the module for implementing the *MMD* function has three *k* × *k* multipliers, four internal registers, and two 2*k*-bit adders. *q*^<*i*>^, which is computed only at the first *j*-iteration, depends of *p*′ and *t*_2_. Once *q*^<*i*>^ is computed, *t*_1_ and *t*_4_ could be computed in parallel. Finally, the partial results are added to obtain *A*^<*i*+1>^. The dataflow from and to the memory blocks is orchestrated by a control module realized as a finite state machine.

### Hardware architecture for MPL

The hardware module for *MMD* in Fig 2 is used to construct the hardware architecture for the MPL algorithm. The inputs of the *MMD* module are the digits *X*_*j*_, *Y*_*i*_, *p*_*j*_, *p*′, and the *A*_*j*_ digits of the partial multiplication *A*^<*i*>^.

Consider the case *e*_*i*_ = 1 in the execution of Algorithm 3. The operand *Y* is the two inputs to the *MMD* multiplier at line 6. Thus, digits from *Y* are read at the outer (*Y*_*i*_) and at the inner loop (*Y*_*j*_) of Algorithm 6. The same applies to *X* when *e*_*i*_ = 0. Therefore, we considered dual port memories when designing the MPL architecture to store and access digits from *X* and *Y* to execute Algorithm 3.

The *MMD* hardware module in Fig 2 now delivers the multiplication result to a memory, and that memory becomes in one Montgomery Multiplier operand at the next iteration. Instead of moving all the content of the memory assigned to *A*^<*i*+1>^ to one of the input memories assigned to *X* or *Y*, our approach is to define a strategy to switch the role of the memories: at one time behaving as an input operand (with read operations) and at another time behaving as the multiplication result (with write operations).

In this context, a total of four memories are required: BRam-*XX*, BRam-*YY*, BRam-*X*, and BRam-*Y*. At the beginning of Algorithm 3, *g* and ‘1’ are loaded into BRam-*X* and BRam-*Y* respectively, and BRam-*XX* and BRam-*YY* play the role of write memories. In the next iteration, the memories change their role, so BRam-*XX* and BRam-*YY* are the input operands and BRam-*X* and BRam-*Y* are now write memories to store the multiplication result in the next iteration. This process continues until all bits of the exponent are processed.

**Fig 3:**
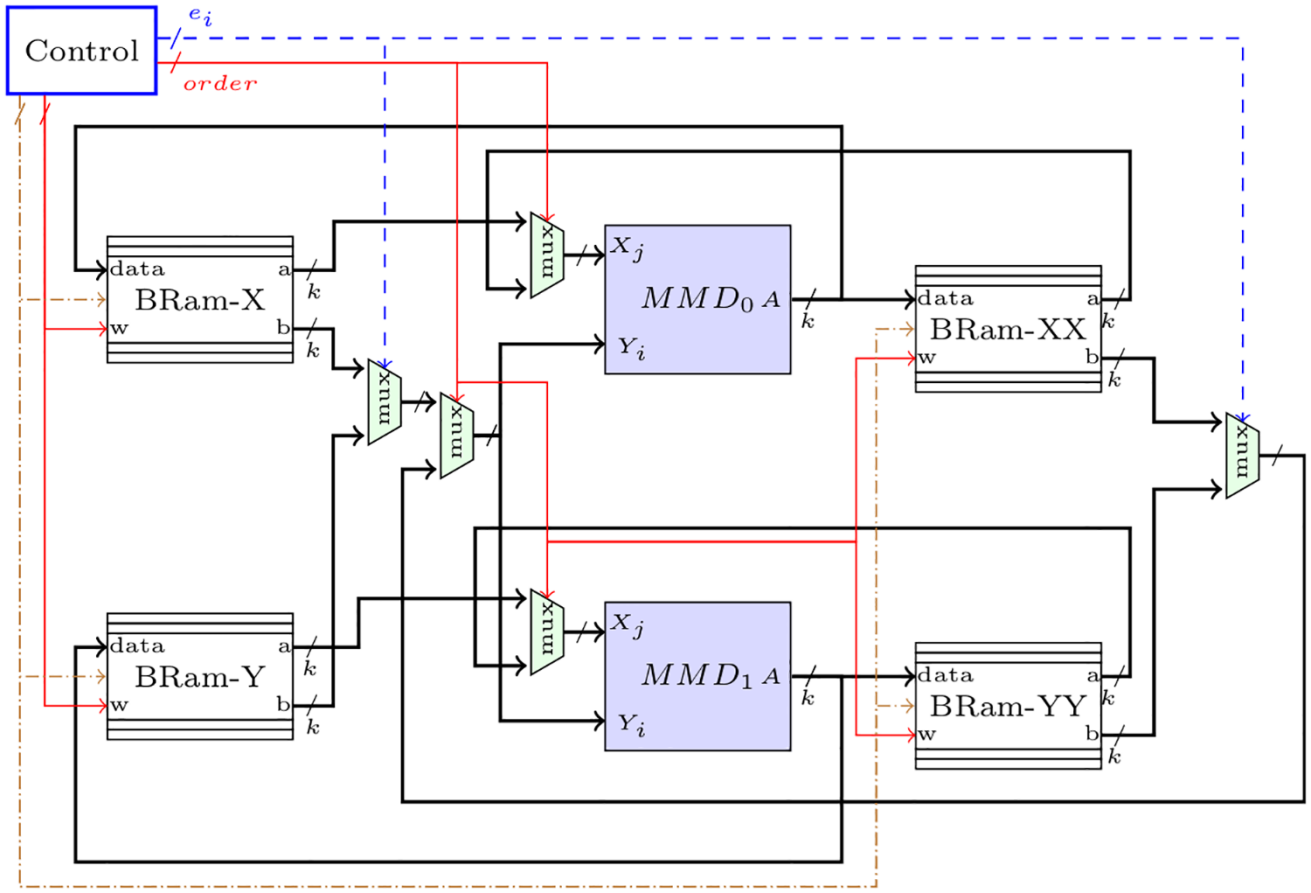
Digit-digit Montgomery Powering Ladder architecture.

The hardware architecture for the MPL algorithm is shown in Fig 3. The main blocks, denoted by *MMD*_0_ and *MMD*_1_, are digit-by-digit Montgomery multipliers executing Algorithm 6. The input ports for these modules are the current input operands at iteration *i* and the output port corresponds to the resulting multiplication delivered digit-by-digit. Other signals such as *p*′, *p* and *A*_*j*_ for *MMD* shown in Fig 2 have been omitted for clarity.

A control unit manages the entire dataflow and stimulates the memory blocks for reading and writing. As we commented before, dual-port memories are used to access two digits at a time from an operand, respectively addressed by the outer and inner loops in Algorithm 6. These two ports are indicated in the block memories of Fig 3 as ‘*a*’ and ‘*b*’.

The proposed hardware architecture presented in Fig 3 takes advantage of available embedded BRams in commercial FPGAs. The exponent *e*, the modulus *p*, and the four temporary variables BRam-X, BRam-Y, BRam-XX and BRam-YY were mapped to FPGA BRams. The exponent *e* and modulus *p* were mapped to single port BRams since only one word per cycle is required. However, the other operands were mapped to dual-port BRams to read from and write to the memory during the same clock cycle. Since all the operands are stored in independent BRams, they can be accessed in parallel without memory bottlenecks. Nevertheless, in the digit-digit multiplication approach only one digit (word) per clock cycle is computed at a time, thus increasing the latency, see Algorithm 6.

Although the reusing of memories saves FPGA resources, the control unit to appropriately stimulate these memories (to read and write digits of operands and partial results) gets more complex. Each memory port requires signals for data input/output, read/write addresses, enable/disable signals, among others. The control unit is in charge of all these signals for orchestrating the algorithm execution and the data flow.

FPGA families have different number of embedded BRams, with a maximum word size. When the word size is bigger than the one allowed, multiple block RAMs are combined to create a single larger RAM. That can increase memory traffic, area and access time due to the interconnections between block RAMs. Because of that, in this work, only word sizes (digit size) of 4, 8, 16, 32, and 64 were implemented.

A relevant aspect of hardware architectures for cryptography applications is their resistance to side-channel attacks. In order to reveal certain secret information when a hardware module perform a encryption/decryption operation, an attacker can perform an analysis of the power dissipation, the electromagnetic radiation, or the operating time of internal operations while the hardware module executes. The Simple Power Analysis (SPA) and Differential Power Analysis (DPA) proposed by Kocher [37] are two of the best known attacks. However, constant time algorithms are resistant to certain side-channel attacks. An deep study about side-channel attacks is presented in [38]. Our proposed Algorithm 6 is a constant time algorithm as the MPL algorithm is. So the proposed algorithms favors the creation of hardware architecture resistant to some side channel attacks such as SPA.

## Implementation results

The hardware architectures proposed in this paper for digit-digit Montgomery multiplication and MPL exponentiation were modeled in VHDL, validated in simulation with Modelsim 10.4, and synthesized for Xilinx FPGAs. The synthesis process was totally automated to generate the configuration bitstreams. During the experimental phase, we use different FPGA families. In order to provide a fairer comparison against related works, we use ISE 14.7 to implement our designs in the Spartan 3, Virtex 5 and Virtex 6 families. However, to provide results with more recent devices, already in use for Industrial IoT applications, we use Vivado V2016.1 to synthesize in the Zynq Z-7010.

The VHDL designs are fully parametrized, so they can be easily configured for different sizes of the digits and operands. The Montgomery multiplier and MPL algorithms were implemented independently for the digit size *k* = 2, 4, 8, 16, 32, 64 bits, and the operand size *N* = 256, 512, 1024, 2048 bits. These operand sizes are currently used in the standard public key cryptosystem RSA. For validation, test vectors were created from software implementations of Algorithms 3 and 6. It is worth to mention that the iterative digit-digit Montgomery multiplication algorithm proposed in [34] uses operands of size less than *N*. That restriction is also kept in this work. In [39] the same algorithm of [34] was adapted to support operands with size less than or equal to *N*.

In this work we also follow one of the approaches in the literature when implementing hardware architectures in FPGAs, the use of embedded IP cores such as DSP modules and Block Rams (BRams). This is generally done to reduce the amount of standard logic of the FPGA, leaving more resources to implement other parts of the security protocol or from the application. Also, this implementation approach allows incrementing the operational frequency and thus improving the execution time and the throughput.

The design and implementation of cryptography hardware architectures in FPGAs depend on the efficient use of architectural features provided in the targeted FPGA. The Xilinx FPGAs used in this work have embedded cores DSPs and BRams which have been employed to reduce the standard logic usage of the proposed design. BRams were used as Dual-Port RAM, and DSP blocks were configured to a multiplier mode. Similar building blocks can also be found in other Xilinx FPGA families such as in the Virtex, Spartan, Kintex, Artix, etc, as well as in the Stratix II and Cyclone II devices of Intel’ FPGAs. So, the proposed technique can be adapted to other FPGAs with similar features. If not fully, our proposed GF(p) exponentiator is highly portable to other FPGA devices.

The metrics used to evaluate the proposed hardware designs are area (slices), performance (bits processed per second—*bps*) and efficiency (*bps* per FPGA slice). Efficiency metric has been used in previous works to evaluate the area resources used and performance achieved in cryptographic hardware architectures [34, 40].

### Digit-digit Montgomery multiplier results

**Fig 4:**
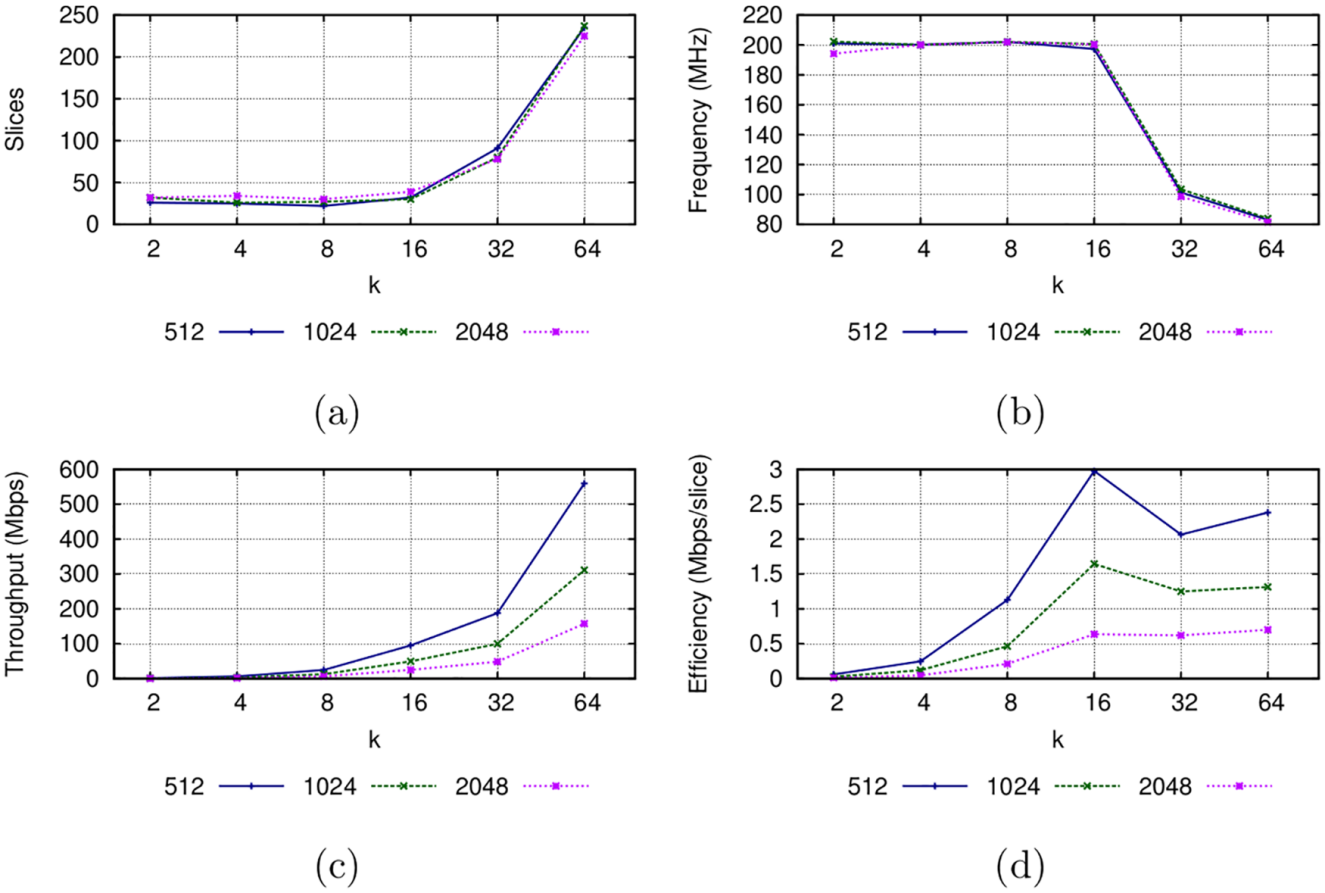
Implementation results of the Montgomery multiplier (Fig 2) in the Virtex-7 FPGA.

The implementation results for the Montgomery multiplier in the Virtex-7 FPGA are shown in Fig 4. The scalability of the proposed multiplier is confirmed with the area results shown in Fig 4a, where it is observed that the size of the operands do not greatly affects the number of slices as the digits do. The best configurations in terms of the use of area are for {*k* = 8, *s* = 256}, {*k* = 4, *s* = 512} and {*k* = 8, *s* = 1024}. When *k* > 16, the needed area increases considerably, possibly due to the interconnections between the CLBs.

The operands size also does not affect the frequency of the multiplier but the digit size does, as it is shown in Fig 4b. This mainly happens because the complexity of multipliers and other components in the datapath increases as the digits get bigger, thus also increasing the critical path in the circuit. A larger operand size will require more digits to process, increasing the latency but not affecting the word size in the datapath or the complexity of the internal hardware modules (adders and multipliers). However, if a greater digit size is used, the latency is reduced. This reduction comes by increasing in the throughput, as Fig 4c reveals. The best result is obtained for *k* = 64, with a throughput of 311.48 Mbps for an operand size of 1024 bits in terms of throughput. Fig 4d reveals that the best efficient Montgomery multiplier is achieved with a digit size *k* = 16 for an operand size of 512 or 1024 bits. When the operand size is 2048 bits, the most efficient multiplier is the one using *k* = 64.

### MPL exponentiator results

**Fig 5:**
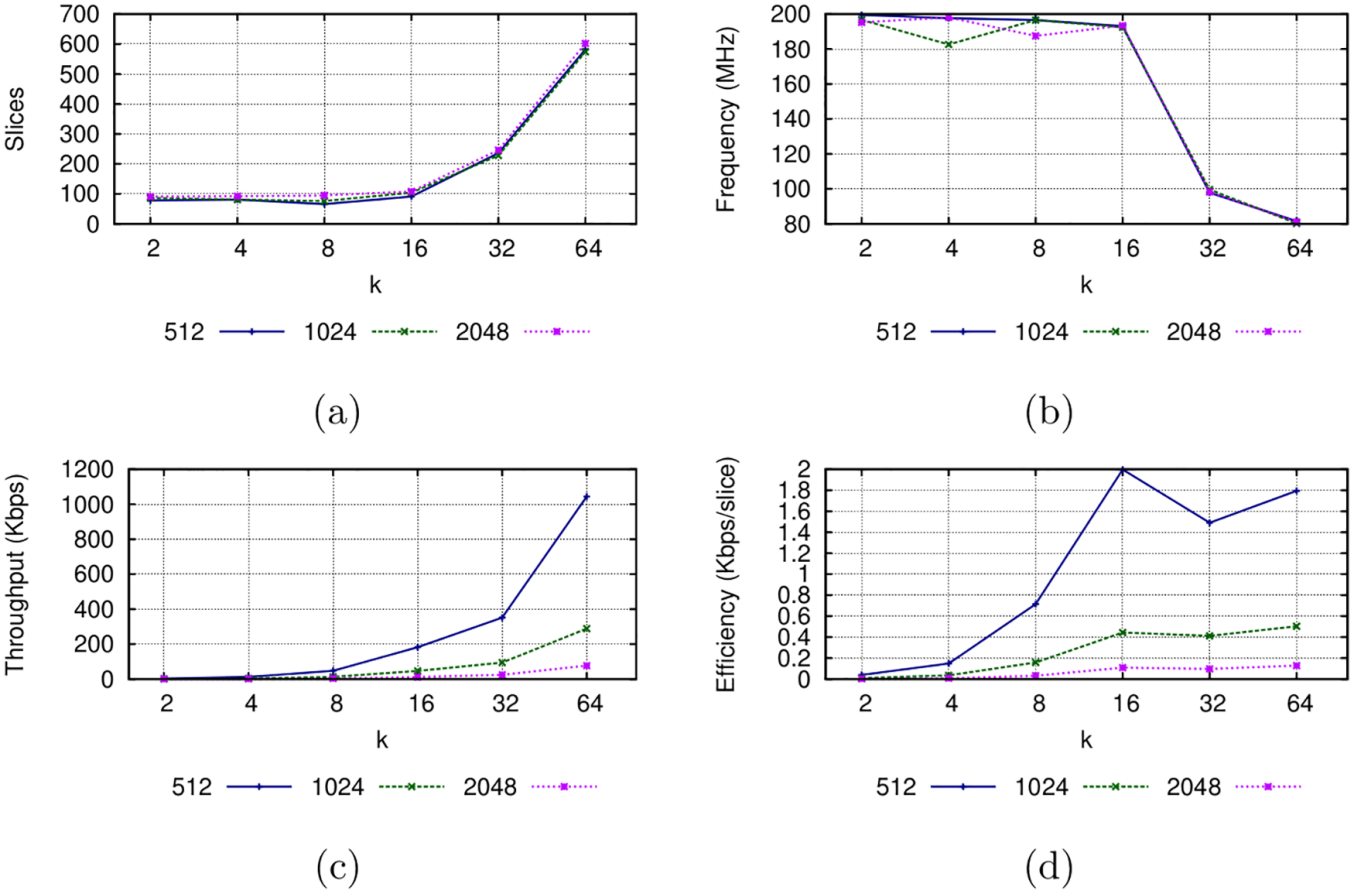
Implementation result for the MPL architecture for a Virtex-7 FPGA.

The implementation results for the Montgomery Powering Ladder architecture are shown in Fig 5. It can be observed that the complexity of the MPL architecture strongly depends on the underlying Montgomery multiplier. For digit sizes from 2 to 16, the area resources remain less than 110 slices. However, the amount of area resources increases considerably when *k* = 32 and *k* = 64. In the same way, the clock frequency remains over 180 MHz when *k* ≤ 16 but degrades considerably when *k* = 32 and *k* = 64, as a consequence of the greater delays due to the use of a larger area. Throughput is considerably reduced, to the order of Kbps, achieving its best for greater digit sizes. In terms of efficiency, considerably better implementations are obtained for greater digit sizes: the best results are for *k* ≥ 16. When *k* ≤ 16 the partial multiplications fit in a single DSP module, but when *k* > 16 partial multiplications in the datapath require several interconnected DSP modules, which increases the number of slices required for interconnection and decreases the frequency.

The results shown in Fig 5 could guide an embedded systems manufacturer to select the most appropriate configuration that allows embedding a hardware encryption accelerator that complies with restrictions on the available area resources, clock frequency, throughput and efficiency.

### Comparison

In this section, a comparison of state of the art GF(p) hardware exponentiation (MPL: Montgomery Powering Ladder, MSB: Most Significant Bit, LSB: Least Significant Bit) is presented. Table 2 shows some of the most significant state of the art works for exponentiation in GF(p). It should be noted that a fair comparison is difficult due to the different technologies and implementation strategies used. It is not possible to compare all the works with the same metric since not all the designs exploit the FPGAs embedded blocks. For example, the digit serial [41] approaches do not use DSPs.

**Table 2:** Results and comparison for a 1024-bit exponentiation.

Work	Alg.	Op.Size (bits)	FPGA	Area (slices)	BRAMs	DSPs	Freq (MHz)	avg Cyc (x 1000)	avg T (ms)	Thrg (Kbps)	Efficiency (kbps/slice)
our.(k = 16)	MPL	1024	Z-7010	**109**	3	6	106.38	4265	40.10	25.535	0.234
our.(k = 32)	MPL	1024	Z-7010	**249**	5	22	68.49	1087	15.76	64.49	0.258
[27]	MPL	1024	Spartan3E	3899	16	20	119.05	946	7.95	128.84	0.033
our.(k = 16)	MPL	1024	Spartan3E	**375**	6	6	77.16	4265	55.29	18.521	**0.049**
our.(k = 32)	MPL	1024	Spartan3E	**900**	6	22	54.59	1087	19.93	51.387	**0.057**
[41](k = 2)	MSB	1024	Virtex-5	7303	-	-	384.62	529	1.38	744.60	0.102
[41](k = 4)	LSB	1024	Virtex-5	6217	-	-	222.11	397	1.79	572.50	0.092
[41](k = 2)	LSB	1024	Virtex-5	4060	-	-	384.62	793	2.03	503.60	0.124
[42]	MPL	1024	Virtex-5	3218	-	-	346.02	1097	3.18	322.01	0.100
[43]	LSB	1024	Virtex-5	6776	-	-	401	-	1.37	747.4	0.110
[43]	MSB	1024	Virtex-5	12716	-	-	401	-	0.92	1113	0.087
our(k = 16)	MPL	1024	Virtex-5	**160**	6	8	190.84	4265	22.35	45.809	**0.286**
our(k = 32)	MPL	1024	Virtex-5	**266**	6	22	73.91	1087	14.71	69.605	**0.262**
[28](k = 16)	LSB	512	Virtex-7	343	-	14	458	-	1.23	416.26	1.214
our(k = 16)	MPL	512	Virtex-7	**91**	6	8	193.12	543	2.82	181.85	**1.998**
[28](k = 32)	LSB	1024	Virtex-7	1060	-	26	485	-	2.33	439.48	0.415
our(k = 64)	MPL	1024	Virtex-7	**574**	10	66	80.21	284	3.55	288.55	**0.503**
[28](k = 64)	LSB	2048	Virtex-7	3558	-	54	399	-	5.68	360.56	0.101
our(k = 64)	MPL	2048	Virtex-7	**602**	10	66	81.11	2174	26.82	76.37	**0.127**

However, we remark here the importance of using the embedded FPGA resources, mainly for efficiency improvement and power saving [24]. The comparison shown in Table 2 is in terms of the standard logic (slices) since the goal of the proposed design is compactness. Although a fair comparison against [41–43] is not possible using slices as metric, it can be done in terms throughput and efficiency. Since [27, 28] also use FPGA embedded resources, a fairer comparison against those works is possible.

The hardware module for MPL implemented in [27] uses the CIOS Montgomery algorithm as GF(p) multiplier. The number of slices is 3899 plus 16 BRAMs, completing an exponentiation in 7.95 ms in an Spartan 3E. Compared to [27], using the same FPGA and operand size of 1024, our design with *k* = 16 is more compact (one-tenth the size), occupying only 375 slices. For *k* = 32, our design still remains with lower area (one-fourth the size), using 900 slices. In terms of efficiency, our design is also better than [27], improving the efficiency by 48% (with *k* = 16) and 72% (with *k* = 32).

The results reported in [41] are among the fastest in the literature, but the FPGA area resources (Virtex-5) consumed are too high, 4060 slices, with an execution time of 2.03 ms. Our design is more efficient than the MPL hardware module reported in [41]. For a 1024-bit modulus, our design with *k* = 16 has an efficiency of 0.286 kbps/slice twice the one achieved by the best version reported in [41].

The hardware module for GF(p) exponentiation reported in [42] for a Virtex-5 FPGA uses 3218 slices, with a throughput of 322.01 kbps and an efficiency of 0.100 kbps/slice. Our design with *k* = 16 uses only 10% of the resources reported in [42] with a better efficiency of 0.286 kbps/slice (more than double).

Our results with the Virtex-5 FPGA can be compared with those of [43]. The best efficiency reported in [43] is 0.110 kbps/slice using an area of 6776 slices. In contrast, our proposed architecture for the same device achieves an efficiency of 0.286 kbps/slice using only 160 slices.

To the authors’ knowledge, the most compact modular exponentiation architecture for FPGAs reported to date is the one presented in [28] for a Xilinx FPGA, using the binary algorithm for GF(p) exponentiation and Montgomery and Karatsuba algorithms for field multiplication. Our design outperforms [28] in terms of efficiency, due to the significant savings in area resources. For a 1024-bit modulus, our design uses half the slices with a better efficiency of 0.503 kbps/slice, and for a 2048-bit modulus, our design is one-sixth the size, as well as having a better efficiency: 0.127 kbps/slice. [28] exploits 17-bit multipliers and 48-bit adder units in DSP blocks to compute the multiplication of high radix integers. The smaller digit size used there is 16, which fits the embedded multipliers in the Xilinx FPGAs. That is why the exponentiation hardware module in [28] cannot be further reduced in size.

The results obtained show that the proposed MPL architecture is smaller than the state of the art in terms of slices, while the number of DSPs and memory blocks required is similar to or less than other works reported in the literature.

Table 3 shows the power estimation generated with Xilinx XPower Analyzer (XPA). Dynamic Powers refers to the quantity and specific use of each resource, and it is considered signals toggling and capacitive loads charging and discharging. So, designs with higher required resources, as well as designs with higher clock frequency will consume more power. Also, big digits require more hardware resources, and as a result, more power consumption. So, in low power devices, it is preferably smaller hardware architectures. On the other hand, quiescent power (also called static power) is not affected by the activity of the design. For example, in Table 3 quiescent power is the same for all configurations. When small digits are used, BRams consume most of the power. However, when bigger digits are used, signals and DSPs require similar power than BRams.

**Table 3:** Supply power (W) of the MPL architecture.

Size	k	Clocks	Logic	Signals	BRAMs	DSPs	IOs	Dynamic	Quiescent	Total
1024	8	0.005	0.003	0.008	0.021	0.006	0.007	0.049	0.178	0.227
1024	16	0.007	0.004	0.012	0.017	0.008	0.013	0.061	0.178	0.239
1024	64	0.006	0.015	0.032	0.036	0.023	0.021	0.132	0.178	0.311
2048	16	0.007	0.004	0.015	0.021	0.008	0.013	0.069	0.178	0.247
2048	64	0.006	0.014	0.029	0.036	0.023	0.021	0.128	0.178	0.307

Although a high throughput is not the aim of the exponentiation architecture proposed in the present paper, it is worth noting that the throughput achieved by our design is better than representative software implementations, as is shown in Table 4. For example, our proposed architecture in Virtex-7 is 600 times faster than the timing achieved in [44], which is aimed at Wireless Sensor Network (WSN) applications.

**Table 4:** GF(*p*) exponentiation in software vs. proposed MPL compact hardware architecture.

Ref.	Imp.	Time
[45]	MSP430 @ 8MHz	≈3 s
[46]	ATmega128 8MHz	10.99 s
[47]	WSN Software	22.03 s
our(k = 64)	Virtex-7	3.55 ms
our(k = 32)	Virtex-5	14.71 ms
our(k = 32)	Zynq-Z7010	15.76 ms

The MSP430 and ATmega128 are two processors commonly used for sensor network research. The proposed design in the Zynq-7010 is 190x faster than the MSP430 implementation, and 697x faster than the ATmega128 implementation. This comparison is only provided to show that the proposed architecture is faster than the software implementations, and to show the proposed hardware accelerates the multiplication and exponentiation in prime fields even using fewer area resources that other hardware implementations in the literature.

These results demonstrate that our proposed design could be used as a small, high-performance hardware accelerator for security in embedded systems.

### In-circuit verification

**Fig 6:**
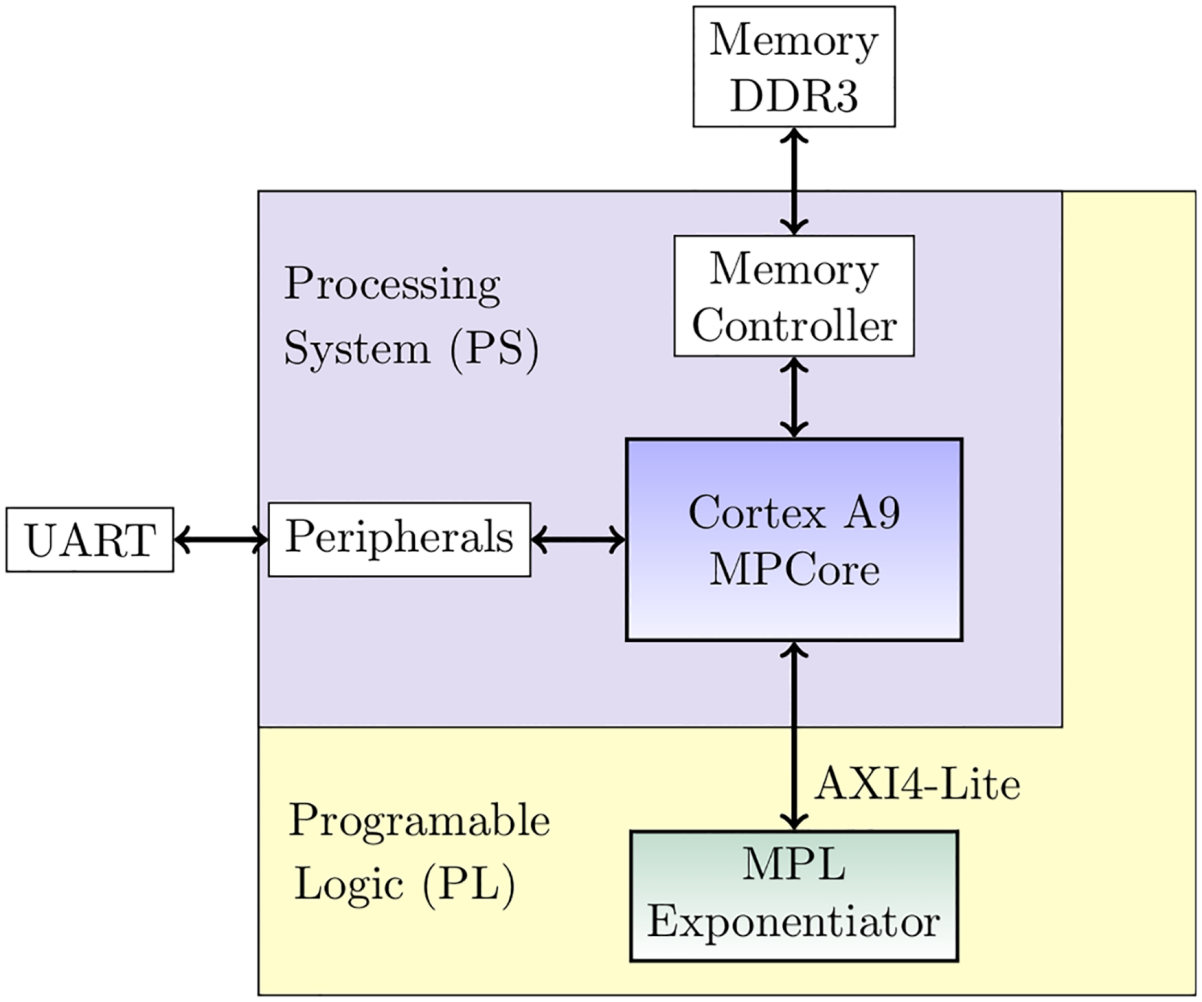
Proposed hardware-software co-design for in-circuit verification of the MPL exponentiator hardware architecture in the Zynq Z-7010 MicroZed.

We carried out an in-circuit verification of our GF(p) exponentiation module by means of a hardware-software co-design (see Fig 6). Under this context, the GF(p) exponentiator is used as a coprocessor commanded by a general purpose processor via a bus interface. The co-design was implemented in the Zynq 7000 SoC family device which combines ARM dual-core Cortex-A9 MPCore processing system (PS) and 28 nm Xilinx programmable logic (PL) in a single device.

The interconnection between PS and PL is a critical aspect since the overhead due to data transfer has a crucial impact in the execution time [28]. We use the AXI4-Lite interface as the communication bus because of its lightweight and area-efficient characteristics if compared to other version such as the AXI4 and AXI-Stream, which are more generally used for high performance designs.

The MPL hardware module was configured to receive data from the PS in words of 32 bits, and fill FPGAs BRams with the operands *p*, *g*, *e* and 1 (one in Montgomery domain). Once BRams are filled with the operators the exponentiation starts. Once the exponentiation is computed the *done* flag is raised up. At the end of the computation, the MPL architecture sends the result in words of 32 bits to the PS. Finally, the PS receives the partial results, merges them and rebuilds the final result. Test vectors were created with the Java API (BigInteger) and used to validate the proposed system-on-chip.

The resources used in the hardware-software co-design are shown in Table 5. The modular exponentiation architecture was configured for a 1024-bit operand size and 32-bit digit size. With this configuration the PL was configure to run at 65 MHz to meet the timing constraint shown in Table 2. These results are provided just as a reference.

**Table 5:** Area usage of the hardware-software co-design implementation in the MicroZed board.

Resource	Used	Available	Utilization (%)
Slices	459	4400	10.43
DSP48E1	22	80	27.50
RAMB36E1	4	60	6.67
RAMB18E1	2	120	1.67

The hardware-software co-design implementation consist of four modules: Zynq PS, PS Reset, AXI Interconect and the proposed MPL architecture. The MPL hardware architecture for 1024 bits operand size with a digit size of 32 require 249 slices as shown in Table 2. However, to connect the coprocessor with the Zynq-7000 AXI interface was added to the MPL incrementing the area resource to 310 slices. The AXI Interconect modules requires 151 slices, and the PS Reset only 7 slices.

Table 6 summarize the power consumption for the proposed SoC implementation. In descending order, the Zynq PS is the module that consumes the most of the power (89.46%). DSPs, BRAMs and Signals are the next most time power consuming and finally the logic and clocks are the components with the least power consumption. Again, these results are presented just as a reference, to serve as a comparison baseline for further research.

**Table 6:** Supply power (W) for the SoC in the MicroZed board.

	Power (W)
Clocks	0.004
Signals	0.010
Logic	0.006
BRAM	0.013
DSP	0.013
Zynq PS	1.529
Dynamic	1.575
Device Static	0.134
Total On-Chip Power	1.709

## Conclusions

Embedded systems in areas such as the medical, military, and surveillance sectors demand secure, low-power and small sized security modules that provide the security services required in networked and pervasive environments. Public key encryption is a useful tool to provide those security services, particularly authentication, integrity and non-repudiation. The present paper addressed the design and implementation issues of a low-area hardware cryptographic module to support the most time consuming operation in public key cryptosystems, exponentiation in prime fields GF(p).

Our design goal was to achieve a low-area hardware architecture suitable to be used as an accelerator of cryptographic operations in embedded systems with reduced computing resources, as typically found in pervasive computing environments. The approach to achieve a low-area design is to process the operands digit-by-digit. The results presented in this paper allow selecting the most appropriate configuration {digit size, operand size} for the exponentiation module to meet specific application requirements of the available area resources, clock frequency, and expected throughput. In general, the most efficient designs for GF(p) exponentiation were obtained for *k* = 16 and *k* = 64.

The proposed design for GF(p) exponentiation uses one-half to one-tenth of the FPGA resources needed by the existing methods in the literature. Thus, more resources are available for implementing other modules because GF(p) exponentiation is only a part of a complete security scheme. So, the MPL architecture is a functional cryptographic module that can be used as a coprocessor in the implementation of cryptographic primitives, such as digital signature in embedded systems.

Without loss of efficiency, our design allows a better usage of FPGA’s slices and at the same time outperforms the running times of GF(p) exponentiation in software implementations.
